# Hygienic and Therapeutic Relations of Good Teeth

**Published:** 1863-03

**Authors:** 


Hygienic and Therapeutic Relations of Good Teeth —Among the most remarkable of the improvements in medical art within the last twenty years, may be mentioned those based on a better understanding of the philosophy of nutrition. Physiology shows that man is an omnivorous animal—that not only can he subsist on both vegetable and animal food, but that perfection requires that he should employ both. Exceptional cases may be brought forward, it is true, but every physiologist is able at once to show that this does not contravene the general law. The man whose system requires meat alone, or vegetable alone, whether this condition be either a constitutional or acquired one, is not in a normal state of health.

Physiology also demonstrates that every particle of food introduced into the stomach should first undergo certain chemical or other changes, by being first thoroughly intermingled with the saliva. It is true, life may be sustained indefinitely where this does not take place; but perfection of health can not be present It is not sufficient to wash down food received with other fluids—it is the sole use of the saliva to facilitate the swallowing process; it is a prominent and necessary digestive fluid. A long train of digestive and other disturbances can be traced directly to neglect of this simple truth—disturbances which the physician may palliate and temporarily remove by his medicines, but which can not cure until the cause is obviated. Dyspepsia, hypochondria, hysteria, gloominess of spirits, neuralgia of all locations, and the catalogue might be long extended, are often dependent on this cause alone—baffling medicines and *pathies* of every hue.

Partly from business haste, partly from habit, often from defective teeth, patients "bolt" their food in half-chewed or solid masses, and when the physiological punishment comes, they repair to a physician, giving their order with similar haste, that he shall remove the effect instantly, so that they can go on in the same evil course, until another cry of indignation comes up from the offended organs of the system. It is easier for the physician to give pill, powder, and mixture than to remonstrate with the patient. It is easier for the patient to swallow them all than to reform. The wagoner, with his mired wheels, prays for Hercules to help him out. The man whose vital wheels are clogged implores the aid of Æsculapius. It were better for both to keep the well surveyed high road. The most that either Hercules or Æsculapius can do, is to get them once more upon the right track.

Bad habits of eating, the patient himself must correct; and the dentist must correct the teeth, so that a thorough mastication and insalivation of food received may secure the prime element of healthy digestion, without which there can be neither continuance of health, or removal of disease.—*People's Dental Journal.*

M. BURGGRAEVE has put into practice the theory of M. Polli—the employment of the sulphites in supposed cases of morbid ferments in the blood. The iatro-chemists, therefore, are still alive, notwithstanding Dr. Chambers's objections to them. M. Burggraeve has communicated to the Belgian Academy of Medicine his experience in the use of these agents in cases of wounds, abscesses, and burns. The sulphite of magnesia is administered internally—one *gramme* (fifteen grains), in a glass of sugared water, four or six times a day. The sulphite of soda is employed externally, in lotions, etc. It produces, we are told, immediate local anesthesia, which is particularly appreciated in burns, and allows of their being dressed and cauterised without pain. In sixty-five cases of wounds thus treated, the effects produced were immediate; the wounds improved, and became of a healthy color; active granulation took place; the pus was scanty, inodorous, and tough as gluten. The application thus also acted as a disinfectant.—*Brit. Jour.*

